# MTA1 promotes metastasis of MPM via suppression of E-cadherin

**DOI:** 10.1186/s13046-015-0269-8

**Published:** 2015-12-21

**Authors:** Caihua Xu, Fei Hua, Yihuan Chen, Haoyue Huang, Wenxue Ye, Yunsheng Yu, Zhenya Shen

**Affiliations:** Department of Cardiovascular Surgery of the First Affiliated Hospital and Institute for Cardiovascular Science, Soochow University, Suzhou, 215000 China

**Keywords:** MTA1, E-cadherin, Malignant pleural mesothelioma, Metastasis

## Abstract

**Background:**

Metastasis-associated gene 1(MTA1) has been identified as an oncogene in many tumors, and aberrant MTA1 expression has been linked to carcinogenesis and metastasis. We aim to investigate the mechanism of MTA1 and metastasis in malignant pleural mesothelioma (MPM).

**Methods:**

Real-time polymerase chain reaction (PCR) and immunohistochemical staining were employed to detect MTA1 and E-cadherin expression in MPM tissues and corresponding adjacent tissues. Stable clone with knock-down of MTA1 was generated with shRNA via lentivirus technology in MPM cell lines. Wound-healing assay, transwell assay and PCR array were carried out for detecting invasion and migration of MPM cells. Luciferase reporter assay was performed to validate the effect of MTA1 on E-cadherin.

**Results:**

MTA1 expression is up-regulated in MPM and shown a negative correlation with E-cadherin expression. MTA1 could enhance the invasion and migration of MPM cells via suppressing the expression of E-cadherin. MTA1 overexpression is associated with pathology, metastasis and survival rate of MPM patients.

**Conclusions:**

MTA1 plays an important role in Epithelial-to-mesenchymal transition (EMT) to promote metastasis via suppressing E-cadherin expression, resulting in a poor prognosis in MPM. MTA1 is a novel biomarker and indicative of a poor prognosis in MPM patients.

**Electronic supplementary material:**

The online version of this article (doi:10.1186/s13046-015-0269-8) contains supplementary material, which is available to authorized users.

## Background

Malignant pleural mesothelioma (MPM) is considered as one of the highest aggressive tumor arising from the cells lining serosal cavities, mostly resulting from the occupational exposure to asbestos fibers [1]. Although great efforts have been made toward improving diagnosis and treatment [2], there are no efficacious therapies for MPM patients presently, and therefore the overall survival is extremely poor. Thus, the investigation of developing the novel therapeutics, especially molecular targeting therapy, is very important for the patients with MPM.

MTA (metastasis-associated gene) is a newly discovered family of cancer progression-related genes and their encoded products. MTA are integral parts of nucleosome remodeling and histone deacetylation (NuRD) complexes, function as transcriptional co-repressors which regulate varieties pathways, including hormonal action, epithelial-to-mesenchymal transitions, differentiations, protein stability and development [3, 4]. MTA1, the first gene found in this family, has been repeatedly reported to be overexpressed along with its protein product MTA1 in a wide range of human cancers such as endometrial adenocarcinomas, gastrointestinal carcinoids, colorectal carcinomas, hepatocellular carcinomas and non-small cell lung cancers [5–10]. However, the potential prognostic relevance of MTA1 expression in MPM has not yet been investigated.

In this study, we aim to investigate the role of MTA1 in the pathogenesis of MPM and identify MTA1 could promote the metastasis of MPM cells by repressing the expression of E-cadherin.

## Methods

### Patients and tissue samples

MPM and corresponding adjacent tissues employed in this study were obtained from 65 consecutive patients who had de novo disease and undergone surgical resection. They were included between December 2008 and November 2013 at the First Affiliated Hospital of Soochow University (Suzhou, China) and the First Affiliated Hospital of Nanjing Medical University (Nanjing, China). The patients were followed up for a median period of 12 months (range, 3–28 months) after operation and their complete clinical data were collected. The correct diagnosis was assessed by an experienced pathologist and the staging of MPM by a clinical oncologist according to AJCC/UICC Guidelines version 7.2010 MPM. Adjacent tissue was located within 3 cm of the edge of the tumor tissue (Additional file 1: Fig. 1S). The study was approved by the Ethical Committee of the First Affiliated Hospital of Soochow University (Suzhou, China) and the First Affiliated Hospital of Nanjing Medical University (Nanjing, China), and written informed consent was obtained from the each patient.

### DNA and RNA preparation

Total RNA was extracted from fresh frozen tissue specimens using TRIzol method (Invitrogen, Shanghai, China) and RNA quality was detected by NanoDrop 2000 and A260/A280 was between 1.95 and 2.05. cDNA was synthesized using reverse transcriptase kit (TAKARA, Tokyo, Japan) according to the manufacturers’ protocol.

### Real-time PCR analysis

MTA1 and E-cadherin mRNA levels were measured by real-time PCR using SYBR Premix Ex Taq (TAKARA, Tokyo, Japan). MTA1 and E-cadherin transcription values were normalized against the expression of β-actin. Amplification conditions, primers, and probes sequences for MTA1 and β-actin were from the work by Zhu X et al. [9] and for E-cadherin were the same as those in the work by Martínez-Estrada et al. [11]. All procedures are in agreement with MIQE guidelines.

### Cell culture

MSTO-211H, NCI-H2452 and 293 T cell lines (ATCC, Manassas, VA) were employed for the present study. MSTO-211H and H2452 were origin from the patients with mesothelioma and cultured in RPMI 1640 medium supplemented with 10 % fetal bovine serum (Invitrogen, Carlsbad, CA), while 293 T were origin from human embryonic kidney cells and cultured in DMEM high glucose medium supplemented with 10 % fetal bovine serum. All cells were maintained in a humidified 37 °C incubator with 5 % CO_2_.

### Lentivirus production and transduction

To generate plasmid-expressing MTA1-shRNA, double-stranded oligonucleotides were cloned into pLL3.7 vector (gifted by D. Yun Chen, Nanjing Medical University, China) and named pLL3.7-shMTA1. The sequences of MTA1-shRNA used are ccggtGACCACCGACAGATACGTG ttcaagaga CACGTATCTGTCGGTGGTCTTTTTTg. The uppercase letters represent MTA1-specific sequence, and lowercase letters represent hairpin sequences. Recombinant lentivirus was generated from 293 T cells using calcium phosphate precipitation. MSTO-211H and H2452 were transfected with lentivirus using polybrene (8 μg/ml).

### Western-blotting assay

Proteins were extracted from cultured cells, quantitated using a protein assay (bicinchoninic acid [BCA] method; Beyotime, Shanghai, China). Proteins were fractionated by sodium dodecyl sulfate polyacrylamide gel electrophoresis, transferred to polyvinylidene fluoride (PVDF) membrane, blocked in 4 % dry milk at room temperature for 1 hour, and immunostained with primary antibodies at 4 °C over-night using anti-MTA1 (1:2000, Abcam, Cambridge, MA), anti-E-cadherin (1:1000; Abcam, Cambridge, MA), and anti-GAPDH (1:1000, Kangchen,China). The results were visualized via a chemiluminescent detection system (Pierce ECL Substrate Western blot detection system; Thermo, Rockford, IL) and exposed in Molecular Imager ChemiDoc XRS System (Bio-Rad, Hercules, CA).

### Cell proliferation assay

Cells were seeded into 96-well plates (6.0 × 10^3^ cells per well). Cell viability was assessed by cell-counting kit-8 assay (CCK-8, Beyotime, Shanghai, China). The absorbance of each well was read on a spectrophotometer (Thermo) at 450 nm (A450). Five independent experiments were performed in quintuplicate.

### Wound-healing assay

Cells were seeded in six-well plates and cultured to confluence. Wounds of 2-mm width were created with a plastic scriber and the floating cells were washed away thrice with phosphate buffered saline (PBS). After incubation in a serum-free medium for 48 hours, cultures were observed and photos were taken under a microscope. A minimum of five randomly chosen areas was measured.

### Transwell invasion assay

The invasive ability of the cells was investigated using Transwells (8-μm pore size; Corning Costar Corp, Bedford, MA) put into the 24-well plates. First, 50 μl Matrigel (50 μg/ml; BD Biosciences, San Jose, CA) was added onto each surface of the chamber, incubated for 2 hours for solidification, then the supernatant was washed away with warm PBS. MSTO-211H and H2452 were suspended in RPMI 1640 containing 2 % fetal bovine serum. A total of 100 μl of the cell suspension (5 × 10^4^ cells) was added to the upper chamber coated with Matrigel, and 400 μl of RPMI 1640 containing 10 % fetal bovine serum was added to the lower compartment. After incubation for 48 hours at 37 °C in a 5 % CO_2_ humidified incubator, the Matrigel and cells on the upper surface of the filter were removed with cotton swabs and the cells that invaded into the lower surface were fixed with 2 % paraformaldehyde, stained with crystal violet. Then the filters were removed from the chambers, air-dried on the precleaned slides and applied with cover-slides using resina. Images were taken under an inverted microscope (Olympus Corp, Tokyo, Japan) at × 100 magnifications over three random fields in each well. ImageJ 1.45 s software (National Insititutes of Health, Bethesda, MD) was used for integrated optical density analysis. Each experiment was performed in triplicate.

### RT-PCR array

Total RNA was extracted from MSTO-211H, MSTO-211H-shMTA1, H2452 and H2452-shMTA and reverse transcribed to cDNA. Subsequently, cDNA was amplified by PCR using 23 Super Array PCR master mix (SuperArray Bioscience, Frederick, MD) and then RT-PCR was carried out using the Human Tumor Metastasis RT2 Profiler PCR array (SuperArray Bioscience, Frederick, MD) in an ABI PRISM7900 system (Applied Biosystems, Foster City, CA), according to the manufacturer’s instructions.

### Luciferase report assay

The promoter of E-cadherin was synthesized artificially and cloned into the luciferase reporter vector (Promega, Madison, WI). MSTO-211H and H2452 cells were seeded into 24-well plates and cotransfected with MTA1-siRNA (pooled siGENOME SMART pool MTA1 siRNA [50 nM per well]; Dhamacon, Chicago, IL), and luciferase reporter vectors using Lipofectamine 2000 (Invitrogen, Carlsbad, CA), following the instructions. Zero, 12 and 24 hours after incubation, cells were collected and firefly and Renilla luciferase activity was measured with the dual-luciferase reporter assay system (Promega). All results were gained through three independent experiments.

### TGF-β1 Inducing EMT of MPM cells

The MPM cells were cultured in vitro and induced by transforming growth fact beta1 (TGF-β1, 5 ng/ml) for 0 h, 24 h, 48 h. The morphological characteristics of cells were observed by microscope (Olympus Corp, Tokyo, Japan) and the expression of MTA1 was detected by real-time PCR.

### Immunohistochemical staining

Tissues were fixed in 4 % paraformaldehyde and cut from paraffin block to 5 μm thickness. After dewaxing with xylene and rehydration with a graded series of ethanol, the slides were heated in the autoclave for three minutes using citrate buffer (PH 6.0) and incubated with primary antibody MTA1 (1:1000, Abcam, Cambridge, MA), E-cadherin (1:1000; Abcam, Cambridge, MA) at 4 °C overnight. Blocking serum or antibody dilution buffer was prepared as Negative controls. The primary antibodies utilized were all the same as for Western blot analysis. Photographs were taken by microscope (Nikon, ECLIPSE 50i) and software NIS-Elements v4.0. Average values of integrated optical density (IOD) were obtained from five random fields per slide by using Image-Pro Plus software (v5.0). Every data was detected three times at least.

### Statistical analysis

Statistical analysis was performed using GraphPad Prism (version 5.01; GraphPad Software, Inc, La Jolla, CA) statistical software. The Student’s t test and paired t test were used to analyze significance between independent groups and paired materials, respectively. The correlation test was used to analyze the correlation between MTA1 and E-cadherin. The χ^2^ test was used to test the significance of observed differences in proportions except when the cells size was less than 5 (Fisher’s exact tests). The significance was accepted as p value was less than 0.05.

## Results

### MTA1 expression is up-regulated in MPM and shown a negative correlation with E-cadherin expression

**Fig. 1:**
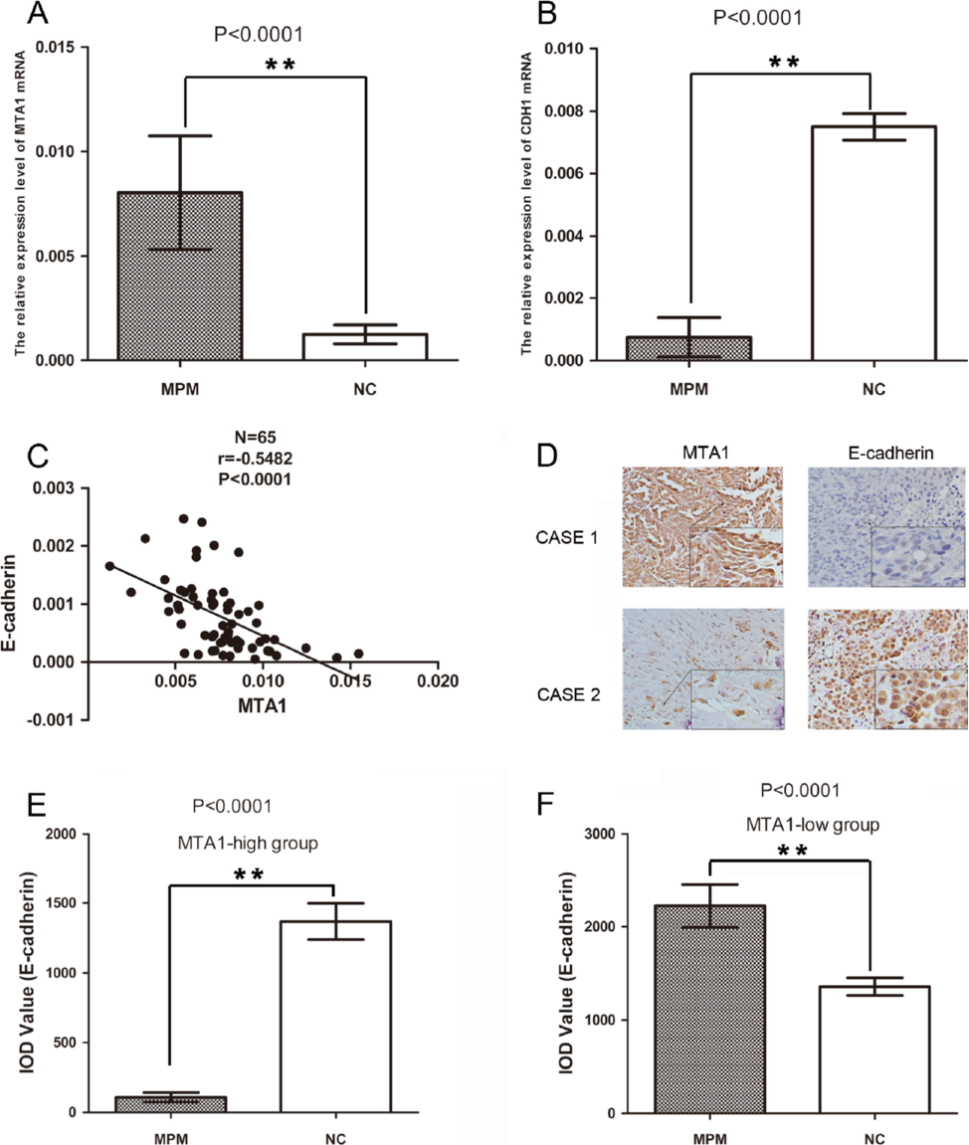
Expression and correlation of MTA1 and E-cadherin transcripts in MPM patients. **a** Significantly higher RNA expression of MTA1 in tumor specimens than in the adjacent tissue samples (*p* < 0.001). **b** Significantly decreased RNA expression of E-cadherin in tumor specimens in comparison with adjacent tissues (*p* < 0.001). **c** A negative correlation between MTA1 and E-cadherin in tumor samples (*r* = −0.5482, *p* < 0.0001). **d** Immunohistochemical staining of E-cadherin in MTA1 overexpression tumor tissue (CASE 1) and MTA1 low expression tumor tissue (CASE 2) in vivo. Data are represented as mean ± SD. *p < 0.05, **p < 0.001. **e** and **f** Average value of integrated optical density (IOD) of E-cadherin in MTA1-high group and MTA1-low group were assessed by analyzing five fields per slide and recorded in the histogram. MTA1, metastasis-associated gene 1; MPM, malignant pleural mesothelioma

We investigated 65 pairs of MPM and corresponding adjacent specimens using real-time PCR and immunohistochemistry. MTA1 RNA level in MPM was significantly higher compared to adjacent tissues (*p* < 0.0001; Fig.1a). However, E-cadherin RNA level was just contrary (*p* < 0.0001; Fig.1b). Moreover, a negative correlation was observed between MTA1 and E-cadherin expression in MPM samples (*r* = − 0.6250; *p* < 0.0001; Fig. 1c). To further evaluate the correlation between MTA1 and E-cadherin expression, two cases with different MTA1 expression levels are shown in Fig. 1d: CASE 1 (strong positive) and CASE 2 (weak positive). The level of E-cadherin was down-regulated in CASE 1 compared to CASE 2. Additionally, statistical analysis of IOD values stained with E-cadherin in MTA1-high group and MTA1-low group are shown in the histogram in Fig. 1e and f (*p* < 0.0001). The results suggested that MTA1 expression was up-regulated and negatively correlated to E-cadherin expression in MPM.

### MTA1 could enhance the invasion and migration of MPM cells in vitro

**Fig. 2:**
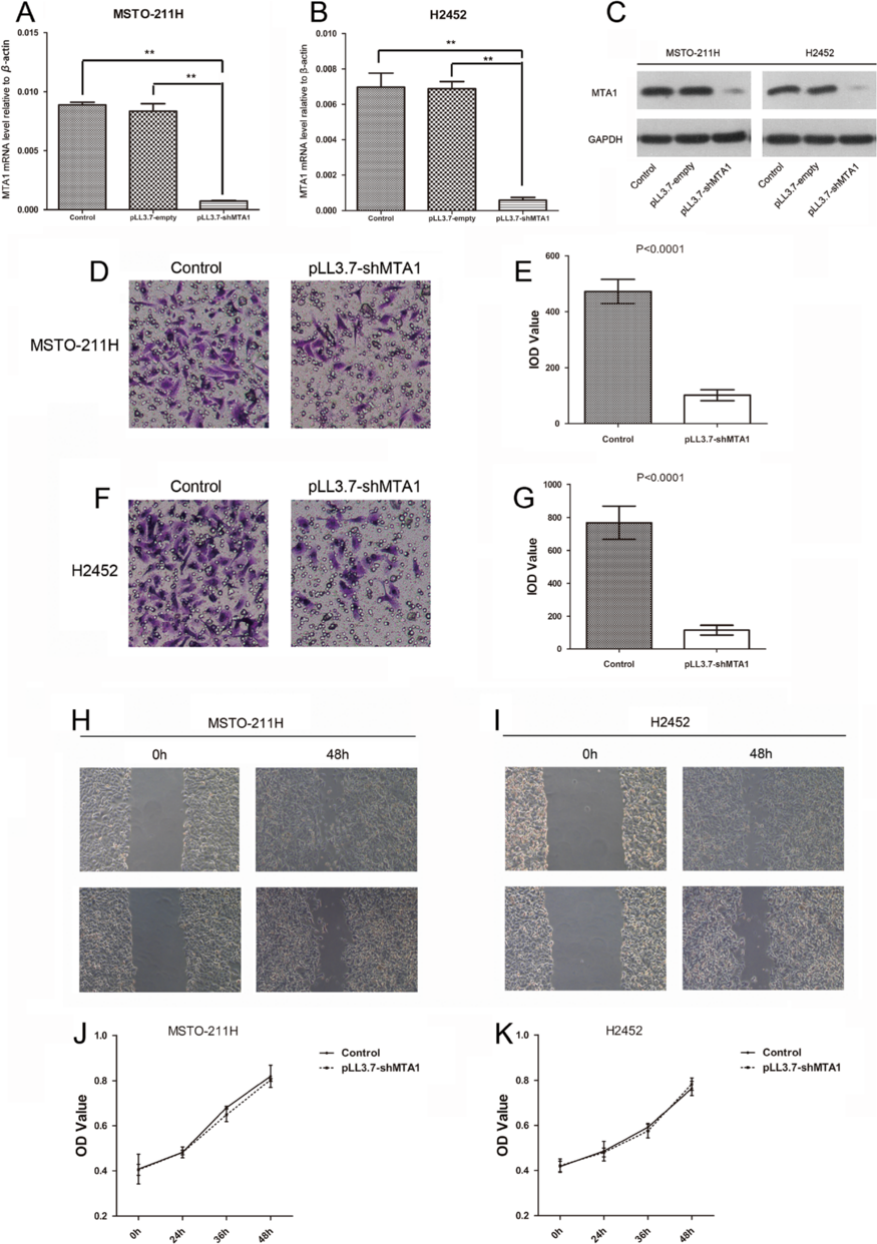
MTA1 enhances the invasion and migration in MPM cells. **a** and **b** MTA1 level of MPM wild-type cells (MSTO-211H and H2452) and MPM cells transfected by lentivirus containing pLL3.7-empty and pLL3.7-shMTA1 by real-time PCR. **c** MTA1 expression of MPM wild-type cells (MSTO-211H and H2452) and MPM cells transfected by lentivirus containing pLL3.7-empty and pLL3.7-shMTA1 by western-blot. **d** and **e** The result of transwell assay showed that the invasion ability of MSTO-211H was significantly suppressed after MTA1 silencing, which was confirmed by integrated optical density (IOD) value evaluation. **f** and **g** *Similar effect of MTA1 was observed in H2452 MPM cells.* **h** and **i** The result of wound-healing assay showed that the migration ability of MPM cells was restrained after MTA1 silencing at 48 h time point. Data are represented as mean ± SD. *p < 0.05, **p < 0.001. **j** and **k**, The viability of MPM wild-type cells (MSTO-211H and H2452) and MPM cells transfected by lentivirus containing pLL3.7-shMTA1 were assessed by CCK-8 assay at 0 h, 24 h, 36 h and 48 h time point. MTA1, metastasis-associated gene 1; MPM, malignant pleural mesothelioma; PCR, polymerase chain reaction; CCK-8, cell-counting kit 8

In vitro, both two MPM cell lines (MSTO-211H and H2452) were transduced by pLL3.7-shMTA1 and detected the expression of MTA1 by real-time PCR and Western-blot (Fig. 2a, b, and c). These results indicated expression of MTA1 was significantly reduced by the lentivirus. As illustrated, the invasion and migration abilities of both MSTO-211H and H2452 were evidently suppressed when MTA1 was down-regulated by pLL3.7-shMTA1 (Fig. 2d, f, h and i). And the quantification of the invasion abilities of MSTO-211H and H2452 are presented in Fig. 2e and g corresponding with Fig. 2d and f. Moreover, We found that MTA1 silencing could not affect cell proliferation (Fig. 2j and k).

### MTA1 knockdown induces increase of E-cadherin expression in MPM cells

**Fig. 3:**
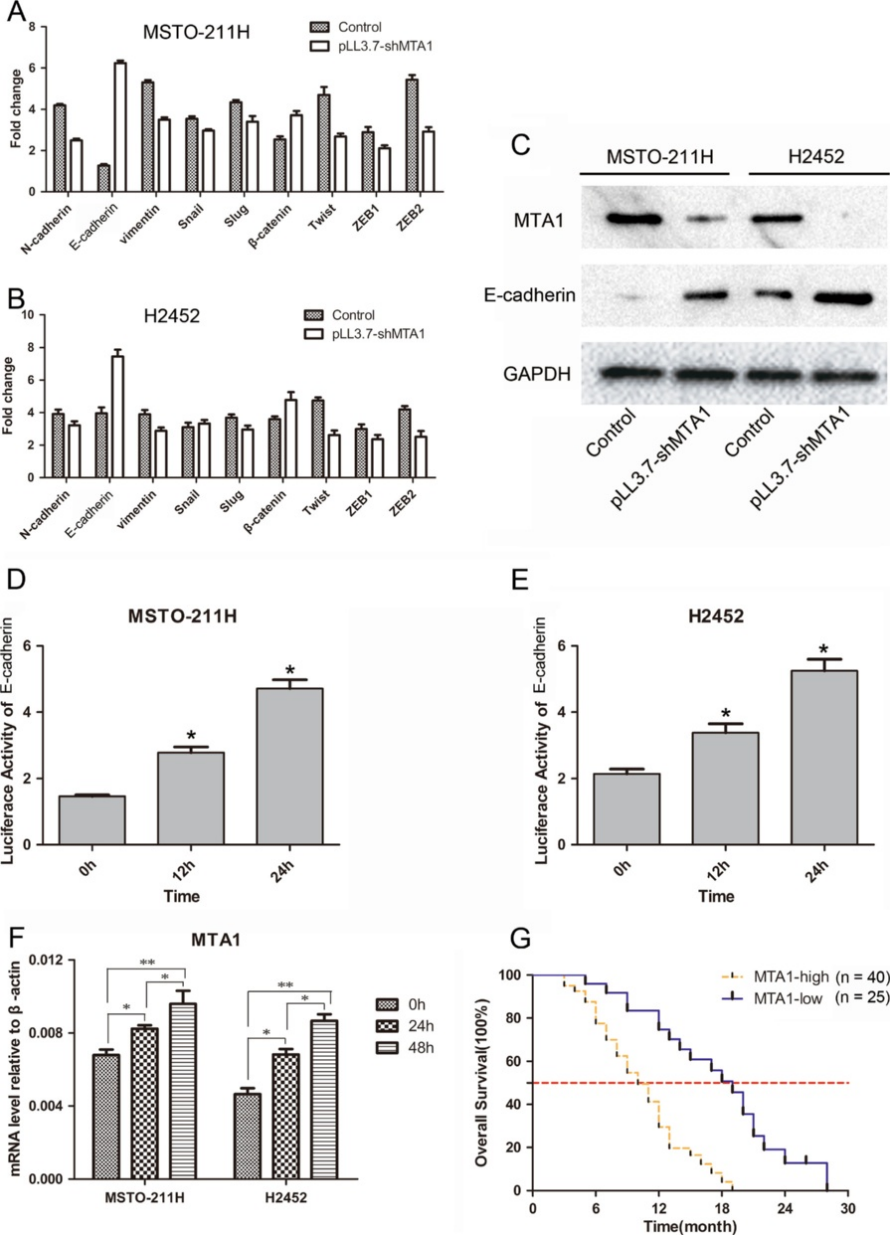
MTA1 suppressed the expression of E-cadherin and was up-regulated by TGF-β1 inducing. **a** and **b** Nine metastasis genes varied larger than two folds were picked via PCR array and were verified by real-time PCR in MPM cells. **c** Western-blot assay validated the result of PCR array of negative trend of MTA1 and E-cadherin in MPM cells. **d** and **e** Luciferase report assay demonstrated that E-cadherin transcription was gradually enhanced in MPM cells transfected by lentivirus containing pLL3.7-shMTA1 in a time-dependent manner. **f** MTA1 mRNA expression was up-regulated in TGF-β1-stimulated MPM cells. **g** MPM patients with a high expression level of MTA1 had a significantly shorter overall survival compared with patients with a low expression level of MTA1. Data are represented as mean ± SD. *p < 0.05, **p < 0.001. MTA1, metastasis-associated gene 1; MPM, malignant pleural mesothelioma; TGF-β1, transforming growth factor beta 1; PCR, polymerase chain reaction

A variety of EMT genes were screened out after MTA1 down-regulation via the PCR array and nine genes whose variation larger than two folds were picked. Then we verified the expression of these nine genes in 65 samples by real-time PCR. We found E-cadherin was up-regulated most after MTA1 silenced in both MSTO-211H and H2452 (Fig. 3a and b). In order to confirm the negative regulatory effect of MTA1 on E-cadherin, we also detected the E-cadherin protein in both two cells by Western-blot. As shown in Fig. 3c, E-cadherin protein expression was increased significantly in both two cells transduced by pLL3.7-shMTA1. Subsequently, we performed luciferase report assay in MSTO-211H and H2452 transfected with MTA1-siRNA and the luciferase activity of E-cadherin was enhanced significantly with transfection time increases (Fig. 3d and e). Moreover, we found the mRNA expression of MTA1 was up-regulated in TGF-β1-stimulated MPM cells (Fig 3f) which was according with the result of Suresh B et al. [12]. Taken together, these findings suggest that the decreased expression of MTA1 could enhance E-cadherin expression in MPM cells.

### MTA1 overexpression is associated with a poor prognosis of MPM

A total of 65 MPM tissues were divided into two groups using a semiquantitative immunoreactivity scoring system (IRS), as reported elsewhere [9, 10, 13, 14]. Concomitant cytoplasmicstaining was not counted. Category A documented the intensity of immunostaining as 0 (no immunostaining), 1(weak immunostaining), 2 (moderate immunostaining), and 3 (strong immunostaining). Category B documented the percentage of immunoreactive cells as 0 (none), 1 (<10 %), 2(10–50 %), 3 (51–80 %), and 4 (80 %). Multiplication of categories A and B resulted in an IRS ranging from 0 to 12 (negative [IRS, 0] versus weak [IRS, 1–4] versus moderate [IRS, 5–8] versus strong [IRS, 9–12]) for each tumor. Both percent positivity of cells and staining intensity were decidedin a double-blinded manner. Interobserver and intraobserver variability was negligible. Tumors with moderate or strong expression (IRS 4) were considered to show MTA1 overexpression, whereas tumors with negative or weak expression (IRS < 4) were considered to show MTA1 down-expression. The relationship of MTA1 expression levels and clinicopathological features were shown in Table 1. Significant higher TNM stage (*p* < 0.01), metastasis (lymph node metastasis and distant metastasis, *p* < 0.01) were observed in high-MTA1 expression group. We also found MTA1 level in sarcomatoid and biphasic malignant mesotheliomas were higher than the level in epithelioid malignant mesothelioma (*p* < 0.05). There was no correlation between MTA1 expression level and sex, age, as shown in Table 1. Additionally, our findings indicated that MPM patients with a high expression level of MTA1 had a significantly shorter overall survival compared with patients with a low expression level of MTA1 and 10 months median survival compared with 19 months median survival after operation (*p* < 0.01, Fig. 3g). These results strongly indicate that MTA1 overexpression contribute to the poor prognosis of MPM patients.

**Table 1:** Correlation of MTA1 expression and clinicopathological features of MPM

Characteristics	All patients	MTA1 expression		*P*	
		Low	High		χ^2^
N	65	25	40		
Sex				0.455	0.559
Male	35	12	23		
Female	30	13	17		
Age (years)				0.842	0.040
<60	27	10	17		
≥60	38	15	23		
Histology				0.038	6.524
Epithelioid	20	13	7		
Sarcomatoid	25	7	18		
Biphasic	20	5	15		
TNM stage^a^				0.003	8.990
I/II	29	17	12		
III/IV	36	8	28		
Lymph node Metastasis				0.001	11.714
Yes	38	8	30		
No	27	17	10		
Distant Metastasis				<0.001	14.562
Yes	35	6	29		
No	30	19	11		

^a^TNM stage was based on AJCC/UICC Guidelines version 7.2010 MPM. MTA1, metastasis-associated protein 1; MPM, Malignant pleural mesothelioma

### Discussion

Malignant pleural mesothelioma (MPM), one of the most deadly human carcinomas, is an aggressive tumor which originates from the mesothelial cells of serosal tissues [15]. Metastasis is the major cause of treatment failure in patients with MPM and its molecular mechanisms are still not clear and are under intensive investigation. To elucidate the mechanisms of metastasis is essential for improving surgery and treatment outcome, especially for selecting and customizing chemotherapy.

In this study, for the first time, we reported that MTA1 gene and protein in MPM specimens was markedly up-regulated compared to adjacent tissues, and MTA1 could enhance the metastasis of MPM cells by direct regulation of E-cadherin. Additionally, the relationship of MTA1 level and clinicopathological features in Table 1 suggests that MTA1 overexpression was associated with the poor prognosis of MPM patients. With all findings taken together, we hypothesized that MTA1 potentially play an oncogenic role in promoting metastasis of MPM.

MTA1 has been considered to be related with the poor prognosis due to increase the migration and invasion of various tumors [6, 8, 16–19]. As a dual function co-regulator, MTA1 operates as a transcriptional repressor of ER-α,BRCA1 and p21 and as a transcriptional activator of BCAS3 and Pax5 [20–24]. Some studies have indicated that down-regulation of MTA1 by RNAi leads to an increase expression of E-cadherin which acts as a key role in epithelial-to-mesenchymal transition in cancer cells [25–27]. Moreover, Wenhao Weng et al. have elucidated that MTA1, together with Snail or Slug, acted to directly repress the promoter activity of E-cadherin in ESCC (esophageal squamous cell carcinoma) cells very recently [28]. Coincidentally, we verified that MTA1 could enhance the epithelial-to-mesenchymal transition by repressing the promoter activity of E-cadherin in MPM cells.

Epithelial-to-mesenchymal transition (EMT) is a cellular process which is considered to be involved in embryo-morphogenesis, fibrosis and tumor metastasis [29, 30]. EMT could enhance not only the ability of migration and invasion, but also the resistance to apoptosis, senescence and drug resistance in cancer [31]. EMT process always accompany by a reduced expression of E-cadherin, cyto-keratins (CK), and β-catenin (in the membrane), and an increased expression of Snail, Slug, Twist, ZEB1, ZEB2, N-cadherin, vimentin, a-smooth muscle actin (aSMA), S100A4, and matrix metalloproteinases (MMP) [31–33]. In particular, repressing the gene expression of E-cadherin could notably promote the EMT which is considered as a primary reason of tumor metastasis. According to our study, the poor prognosis of patients with high MTA1expression was correlated with E-cadherin, the very classical metastasis suppressor.

## Conclusion

Our study identified the negative correlation of MTA1 and E-cadherin in 65 MPM cases, both of which were associated with TNM stage, metastasis and histological types and proved the regulatory function of MTA1 on E-cadherin through direct binding of its promoter. However, as a limit of the number of MPM cases and cell types, more elaborate research will be requireda for further exploration of MTA1 in tumorigenesis and metastasis. Thus, MTA1 potentially served as a therapeutic target for the treatment of MPM.

## Additional file

Additional file 1: Figure 1S. MTA1 protein expression in tumor tissue and adjacent tissue of one MPM patient. A and B, immunohistochemical staining of MTA1 in tumor specimen in comparison with adjacent tissue. (TIF 2636 kb)

## Abbreviation

MTA: Metastasis-associated gene
MTA1: Metastasis-associated gene 1
MPM: Malignant pleural mesothelioma
PCR: Polymerase chain reaction
EMT: Epithelial-to-mesenchymal transition
NuRD: Nucleosome remodeling and histone deacetylation
CK: Cyto-keratins
aSMA: A-smooth muscle actin
MMP: Matrix metalloproteinases
