# Correction: MTA1 promotes metastasis of MPM via suppression of E-cadherin

**DOI:** 10.1186/s13046-026-03750-2

**Published:** 2026-06-23

**Authors:** Caihua Xu, Fei Hua, Yihuan Chen, Haoyue Huang, Wenxue Ye, Yunsheng Yu, Zhenya Shen

**Affiliations:** Department of Cardiovascular Surgery of the First Affiliated Hospital and Institute for Cardiovascular Science, Soochow University, Suzhou, 215000 China

**Correction: J Exp Clin Cancer Res 34, 151 (2015)**

**https://doi.org/10.1186/s13046-015-0269-8**

Following publication of the original article [1], the authors spotted errors in the Fig. 2 image, specifically, wound-healing assay image of H2452 cells infected with pLL3.7-shMTA1 in Fig. 2I was mistakenly used using the image of MSTO-211 H in Fig. 2H.

The correct figure is presented below:

**Incorrect Fig. 2H and I**

**Fig. 2:**
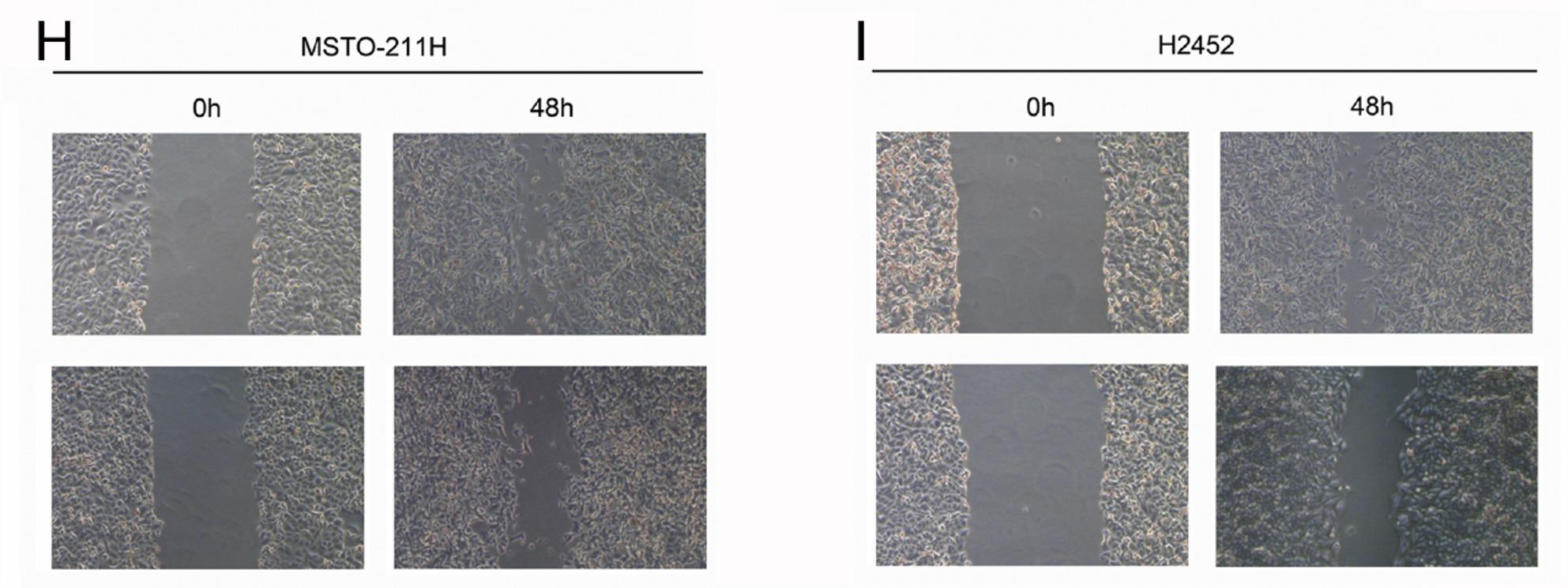
MTA1 enhances the invasion and migration in MPM cells. **a** and **b** MTA1 level of MPM wild-type cells (MSTO-211H and H2452) and MPM cells transfected by lentivirus containing pLL3.7-empty and pLL3.7-shMTA1 by real-time PCR. c MTA1 expression of MPM wild-type cells (MSTO-211H and H2452) and MPM cells transfected by lentivirus containing pLL3.7-empty and pLL3.7-shMTA1 by western-blot. **d** and **e** The result of transwell assay showed that the invasion ability of MSTO-211H was significantly suppressed after MTA1 silencing, which was confirmed by integrated optical density (IOD) value evaluation. **f** and **g** Similar effect of MTA1 was observed in H2452 MPM cells. **h** and **i** The result of wound-healing assay showed that the migration ability of MPM cells was restrained after MTA1 silencing at 48 h time point. Data are represented as mean ± SD. *p < 0.05, **p < 0.001. **j** and **k**, The viability of MPM wild-type cells (MSTO-211H and H2452) and MPM cells transfected by lentivirus containing pLL3.7-shMTA1 were assessed by CCK-8 assay at 0 h, 24 h, 36 h and 48 h time point. MTA1, metastasis-associated gene 1; MPM, malignant pleural mesothelioma; PCR, polymerase chain reaction; CCK-8, cell-counting kit 8

**Correct Fig. 2H and I**

**Fig. 2:**
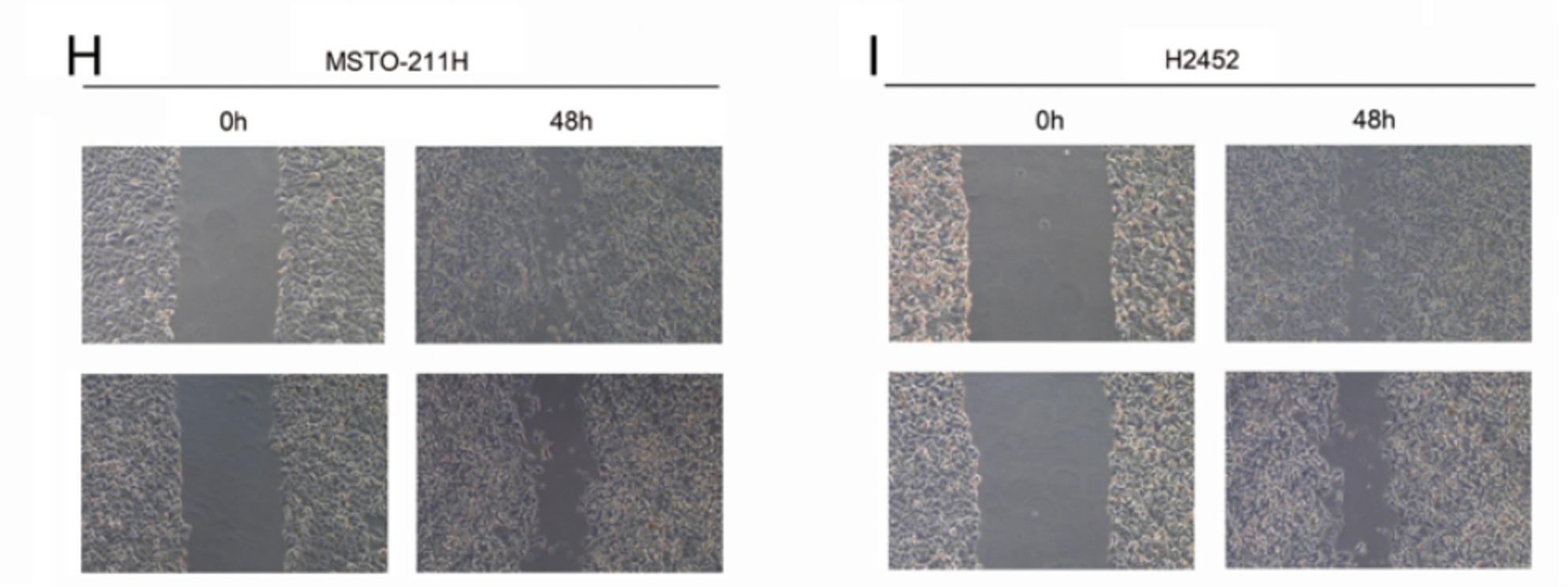
MTA1 enhances the invasion and migration in MPM cells. **a** and **b** MTA1 level of MPM wild-type cells (MSTO-211H and H2452) and MPM cells transfected by lentivirus containing pLL3.7-empty and pLL3.7-shMTA1 by real-time PCR. c MTA1 expression of MPM wild-type cells (MSTO-211H and H2452) and MPM cells transfected by lentivirus containing pLL3.7-empty and pLL3.7-shMTA1 by western-blot. **d** and **e** The result of transwell assay showed that the invasion ability of MSTO-211H was significantly suppressed after MTA1 silencing, which was confirmed by integrated optical density (IOD) value evaluation. **f** and **g** Similar effect of MTA1 was observed in H2452 MPM cells. **h** and **i** The result of wound-healing assay showed that the migration ability of MPM cells was restrained after MTA1 silencing at 48 h time point. Data are represented as mean ± SD. *p < 0.05, **p < 0.001. **j** and **k**, The viability of MPM wild-type cells (MSTO-211H and H2452) and MPM cells transfected by lentivirus containing pLL3.7-shMTA1 were assessed by CCK-8 assay at 0 h, 24 h, 36 h and 48 h time point. MTA1, metastasis-associated gene 1; MPM, malignant pleural mesothelioma; PCR, polymerase chain reaction; CCK-8, cell-counting kit 8
