# Supplementary figures and images for: MTA1 promotes metastasis of MPM via suppression of E-cadherin
Source: J Exp Clin Cancer Res. 2015 Dec 21;34:151. doi: 10.1186/s13046-015-0269-8 (PMC4687136; doi:10.1186/s13046-015-0269-8)

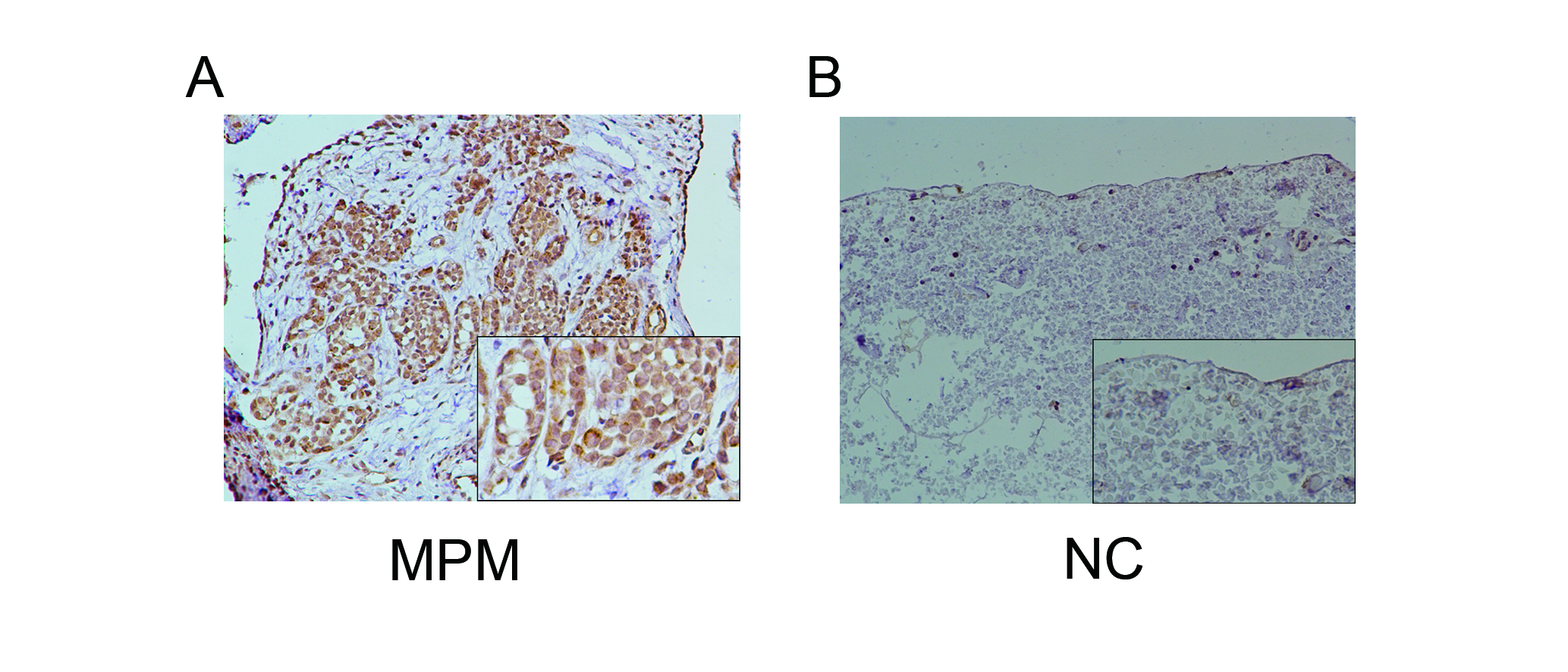

Supplement: Additional file 1: Figure 1S. — MTA1 protein expression in tumor tissue and adjacent tissue of one MPM patient. A and B, immunohistochemical staining of MTA1 in tumor specimen in comparison with adjacent tissue. (TIF 2636 kb) [file 13046_2015_269_MOESM1_ESM.tif]
